# Rasch-based classification of post-stroke balance ability: dimensionality and clinical validation of the BESTest and S-BESTest

**DOI:** 10.3389/fnagi.2026.1889177

**Published:** 2026-10-02

**Authors:** Thitimard Winairuk, Marco Y. C. Pang, Sirod Sirisup, Vitoon Saengsirisuwan, Kamolwan K. Klaykaew, Rumpa Boonsinsukh

**Affiliations:** 1 Faculty of Physical Therapy, Srinakharinwirot University, Nakhon Nayok, Thailand; 2 Department of Rehabilitation Sciences, The Hong Kong Polytechnic University, Hong Kong, Hong Kong SAR, China; 3 Data-Driven Simulation and Systems Research Team, National Electronics and Computer Technology Center, Pathum Thani, Thailand; 4 Department of Physiology, Faculty of Science, Mahidol University, Bangkok, Thailand; 5 Innovative Learning Center, Srinakharinwirot University, Bangkok, Thailand

**Keywords:** cerebrovascular disease, hemiparesis, postural control, psychometric properties, Rasch analysis

## Abstract

**Background:**

The Balance Evaluation Systems Test (BESTest) and its stroke-specific short version (S-BESTest) are widely used to evaluate balance after stroke. However, clinically meaningful classifications of balance ability derived from these instruments remain unavailable.

**Objectives:**

To examine the dimensionality of the BESTest and S-BESTest and establish Rasch-based classifications of balance ability in individuals with stroke.

**Methods:**

In this cross-sectional study, 335 individuals with stroke from two tertiary care hospitals completed the BESTest and S-BESTest. Exploratory factor analysis (EFA) was performed to identify the underlying factor structure of each instrument. Principal component analysis of Rasch residuals (PCAR) and disattenuated correlations between Rasch person measures were subsequently used to evaluate essential unidimensionality. Rasch analysis using the Andrich Rating Scale Model was then conducted to derive balance ability classifications and corresponding raw-score cutoffs. Known-groups validity of the proposed classifications was examined using lower extremity Fugl-Meyer Assessment (FM-LE) scores.

**Results:**

EFA identified a three-factor solution for the BESTest and a two-factor solution for the S-BESTest. However, PCAR and disattenuated correlations demonstrated that both instruments functioned as essentially unidimensional measures of balance ability. Rasch analysis showed acceptable-to-excellent reliability and yielded clinically interpretable balance ability classifications with corresponding raw-score cutoff values. The BESTest was classified into six balance impairment levels, ranging from very severe to very mild impairment, whereas the S-BESTest was classified into four levels, ranging from very severe to mild impairment. FM-LE scores differed significantly across all classification levels for both instruments, supporting good known-groups validity.

**Conclusion:**

Although exploratory factor analysis identified multiple factors, the BESTest and S-BESTest functioned as essentially unidimensional measures of balance ability in individuals with stroke. The proposed Rasch-based classifications provide clinically interpretable balance ability levels that may facilitate communication, facilitate individualized rehabilitation planning, and support clinical decision-making.

## 1 Introduction

The Balance Evaluation System Test (BESTest) is a clinical scale designed to identify the underlying causes of balance impairment and to guide balance training targeted to specific deficits in postural control (Horak et al., 2009). It comprises six components: biomechanical constraints, stability limits/verticality, anticipatory postural adjustments, postural responses, sensory orientation, and stability in gait. The BESTest has demonstrated excellent reliability and concurrent validity, with no floor or ceiling effects in individuals with subacute stroke (Chinsongkram et al., 2014, 2016; Winairuk et al., 2019). However, with 36 items across 27 tasks, administration requires nearly an hour, limiting its feasibility in routine clinical practice. To address this issue, two shortened versions of the BESTest were developed; the Mini-BESTest and Brief-BESTest (Franchignoni et al., 2010; Padgett et al., 2012). The Brief-BESTest contains only 6 items, one for each domain of the BESTest, while The Mini-BESTest omits important domains such as biomechanical constraints and stability limits/verticality. Exclusion of biomechanical measures such as hip and ankle strength reduces the ability to capture functional performance relevant to balance control. Comprehensive assessment across all domains of postural control remains essential for accurate diagnosis and effective intervention planning.

More recently, the Stroke-BESTest (S-BESTest) was developed as a short version of the BESTest for individuals with subacute stroke (Winairuk et al., 2017). Its 13 items were selected from the 36-item BESTest through Rasch analysis to span the range of item difficulty across the six postural control domains. Scoring criteria are identical to those of the BESTest. Subsequent validation of the S-BESTest demonstrated excellent reliability, validity, and responsiveness (Winairuk et al., 2019). Specifically, the S-BESTest has shown excellent intra-rater reliability [intraclass correlation coefficient (ICC) = 0.98] and inter-rater reliability (ICC = 0.95). Concurrent validity was also strong, with high correlations observed with the Berg Balance Scale (*r* = 0.95), the Brief-BESTest (*r* = 0.93), and the Mini-BESTest (*r* = 0.95). No significant floor or ceiling effects were detected (<20%). The S-BESTest demonstrated sufficient internal responsiveness, with large standardized-response-means (SRM) of 1.28 (0–2 weeks after rehabilitation) and 1.29 (2–4 weeks after rehabilitation). Furthermore, it provided greater accuracy than the Brief-BESTest and Mini-BESTest in detecting balance improvements in individuals with subacute stroke, as indicated by a minimal clinically important difference (MCID) of six points (Winairuk et al., 2019).

The BESTest and S-BESTest reliably detect multiple domains of balance impairments in individuals with stroke, including deficits in sensory orientation, adaptive responses, and anticipatory postural adjustment (Winairuk et al., 2019). These impairments are consistent with those reported in previous studies such as abnormal sensory integration associated with reduced activity participation (Chen et al., 2018), shortened step length and duration with multiple corrective steps during adaptive responses (Chayasit et al., 2020), and delayed onset of muscle activity on the affected side during anticipatory tasks (Curuk et al., 2020). However, neither scale provided clinically interpretable classification of balance ability levels, which are important for guiding individualized interventions. The absence of clinically interpretable classifications limits score interpretation, patient stratification, and selection of appropriate intervention intensity in clinical practice. Accurate classification of balance ability level enables clinicians to tailor rehabilitation strategies, monitor recovery progress, and select intervention intensity appropriate to each patient’s level of functional ability.

Rasch analysis enables the estimation of item difficulty and measurement precision while providing clinically interpretable severity classifications along a continuum of functional balance ability in individuals with stroke (Franchignoni et al., 2015; Miyata et al., 2022; Winairuk et al., 2022). Prior to Rasch analysis, exploratory factor analysis (EFA) was performed to examine the underlying factor structure of the BESTest and S-BESTest. EFA is widely used in the development and validation of assessment instruments to identify latent constructs underlying relationships among questionnaire items and to provide evidence of construct validity (Fabrigar et al., 1999; Osborne et al., 2008). Following EFA, principal component analysis of Rasch residuals (PCAR) was further recommended to determine whether the EFA-derived factors represent independent latent dimensions or a single underlying construct (Smith, 2002). Establishing dimensionality using PCAR is particularly important because Rasch modeling assumes that items within each factor represent a common underlying construct (Edelen and Reeve, 2007). To support the clinical interpretability of the proposed balance ability classifications, known-groups validity against an established measure of lower extremity motor ability was also performed. The lower extremity motor domain of the Fugl-Meyer Assessment (FM-LE) is a widely used and well-validated measure of post-stroke motor recovery and may provide supportive evidence for the clinical validity of balance ability classifications. Accordingly, this study aimed to identify the dimensionality of the BESTest and S-BESTest using EFA, establish Rasch-based classifications for balance ability within each identified factor, and examine the clinical validity of these classifications using the FM-LE in individuals with stroke.

## 2 Methodology

### 2.1 Participants

This observational cross-sectional study recruited individuals with stroke from the physical therapy departments of two tertiary care hospitals: Lerdsin Hospital and Prasat Neurological Institute. Sample size was considered adequate for both exploratory factor analysis (EFA) and Rasch analysis. For EFA, recommendations of at least 5–10 participants per item were followed (Osborne and Costello, 2005). With 335 participants, the participant-to-item ratios for both the BESTest and the S-BESTest exceeded these recommendations. Sampling adequacy was further evaluated using the Kaiser–Meyer–Olkin (KMO) measure and Bartlett’s test of sphericity. For Rasch analysis, recommendations indicate that a sample of approximately 250 participants provides stable Rasch item calibrations within ±0.5 logits at the 99% confidence level (Linacre, 1994).

Participants were consecutively recruited according to the following inclusion criteria: (1) first unilateral hemispheric stroke, (2) stable vital signs, and (3) ability to follow verbal commands and complete the assessments. Exclusion criteria were: (1) neurological disorders other than stroke, (2) brainstem or cerebellar lesions, (3) uncontrolled epilepsy, (4) cerebral aneurysm, (5) uncorrected visual impairment, and (6) cognitive impairment, defined as a Mini-Mental State Examination Thai version (MMSE-Thai) score ≤ 23 (Dick et al., 1984; Tombaugh and McIntyre, 1992; Thailand, 2008).

### 2.2 Procedures

Written informed consent was obtained from all participants before data collection. The study was approved by the institutional review boards of Lerdsin Hospital and Prasat Neurological Institute (EC0519.18/085.1 and EC038/2559).

Before testing, participants’ vital signs were assessed to ensure medical stability. Two experienced physical therapists (>5 years of clinical experience in stroke rehabilitation), each stationed at one participating hospital, conducted all assessments. Prior to participant recruitment, intra-rater (ICC = 0.98, 95% CI 0.97–0.99) and inter-rater reliability (ICC = 0.95, 95% CI 0.91–0.98) were established by independently scoring videotaped performances of 12 individuals with subacute stroke on two occasions separated by 7 days without discussion between raters.

Each participant was assessed once by one of the two therapists. Standardized verbal instructions were provided throughout testing, and rest periods were allowed whenever necessary. The BESTest was administered directly and required approximately 1 h to complete. S-BESTest scores were subsequently extracted from the corresponding BESTest items.

### 2.3 Data and statistical analysis

Descriptive statistics were used to summarize participants’ demographic and clinical characteristics.

#### 2.3.1 Exploratory factor analysis

The construct structure of the BESTest and S-BESTest was initially explored using EFA performed in SPSS version 26.0 (IBM Corp., Armonk, NY, USA). Rather than assuming a priori dimensionality, EFA was conducted to identify the underlying factor structure of the BESTest and S-BESTest. These empirically derived factors were subsequently evaluated using Rasch analysis to determine whether they represented distinct latent constructs or reflected a single underlying dimension of balance ability.

The suitability of the data for factor analysis was assessed using the KMO measure and Bartlett’s test of sphericity. KMO values were interpreted according to Kaiser’s criteria as follows: ≥0.90 = marvelous, ≥0.80 = meritorious, ≥ 0.70 = middling, ≥ 0.60 = mediocre, and <0.50 = unacceptable (Kaiser, 1974). Parallel analysis was used to determine the number of factors to retain. Maximum likelihood extraction with Varimax rotation was performed. Items with factor loadings below 0.40 were removed, and the analysis was repeated using the reduced item set. The proportion of variance explained by each factor was estimated using eigenvalues.

#### 2.3.2 Rasch analysis

Rasch analysis was subsequently performed for the overall scales and separately for each EFA-derived factors using the Andrich Rating Scale Model (RSM) implemented in WINSTEPS version 4.0.1 (Winsteps^®^, SWREG Inc., Eden Prairie, MN, USA). The RSM was selected because all BESTest and S-BESTest items employ the same four-category (0–3) response scale representing increasing levels of performance. In contrast to the Partial Credit Model, which allows item-specific threshold structures, the RSM assumes a common category structure across all items (Andrich, 1978; Bond et al., 2021; Linacre, 2002). In addition, inspection of the category frequencies indicated that several of the easiest BESTest items contained fewer than the recommended minimum of 10 observations in the lowest response categories for stable item-specific threshold estimation (Linacre, 2002), further supporting the use of the RSM rather than the Partial Credit Model. The appropriateness of the common rating scale was subsequently evaluated by examining category functioning, including average category measures, Andrich thresholds, category fit statistics, and category probability curves. Ordered category measures and thresholds, together with acceptable fit statistics and category probability curves, supported the functioning of the common rating scale.

Item and person fit to the Rating Scale Model were evaluated using infit and outfit mean-square (MnSq) statistics. Values between 0.5 and 1.5 were considered acceptable for productive measurement, whereas values above 1.5 indicated potential underfit and values below 0.5 indicated potential overfit (Linacre, 2002; Wright and Linacre, 1994). Greater emphasis was placed on infit when evaluating potential misfit because it is more sensitive to responses that are unexpected for persons whose ability levels are close to the item difficulty. Person misfit was quantified as the proportion of participants with outfit MnSq ≥ 2.0 (Linacre, 2002). Participants with extreme scores were excluded from person-fit calculations because fit statistics are not estimated for these cases. No items or participants were removed solely on the basis of fit statistics; potential misfit was evaluated in conjunction with item content and the overall measurement structure.

Following EFA, principal component analysis of Rasch residuals (PCAR) was performed to determine whether the EFA-derived factors represented independent latent dimensions or whether they functioned as components of a single underlying construct (Smith, 2002). Evidence supporting essential unidimensionality included: (1) Rasch measures explaining at least 20%–40% of the total variance, (2) first residual contrast eigenvalues < 2.0, and (3) the absence of meaningful secondary dimensions (Linacre, 2024).

To further examine the relationships among the EFA-derived factors, disattenuated correlations between Rasch person measures were calculated. Disattenuated correlations greater than 0.90 were interpreted as indicating that the item clusters measured essentially the same latent construct rather than independent dimensions (Linacre, 2024).

#### 2.3.3 Rasch-derived balance ability classifications

Rasch-derived balance ability classifications for the overall BESTest and S-BESTest were developed in three steps. First, the maximum number of statistically distinguishable strata was estimated using the formula [(4G) + 1]/3, where G is the person separation index (Wright, 2001; Wright and Masters, 2002). This estimate was used as an upper bound on the number of ability levels supported by the measurement precision rather than as a prescription for the final number of clinical categories. The formula is based on the definition of statistically distinguishable strata as ability levels separated by approximately three standard errors of measurement (SEM) (Wright and Masters, 2002). Second, candidate classification boundaries were established at regular raw-score intervals. Regular raw-score intervals were selected because both instruments are administered and interpreted using raw scores and because such boundaries facilitate practical clinical application. Third, candidate boundaries were verified using the Rasch raw score-to-measure conversion table. For each candidate category, the person measure and its standard error at the midpoint score were obtained, and the difference between adjacent midpoint measures was expressed in units of the mean SEM. Candidate categories were considered statistically distinguishable when adjacent category midpoints differed by more than 3 SEM, with person measures also increasing monotonically across categories.

#### 2.3.4 Floor and ceiling effects

Floor and ceiling effects were examined to evaluate potential limitations in score distribution. Floor effects were defined as the proportion of participants obtaining the minimum possible score, whereas ceiling effects represented the proportion achieving the maximum possible score. Effects ≥20% were considered substantial (Portney, 2020).

#### 2.3.5 Known-groups validity

Known-groups validity of the Rasch-derived balance ability classifications was evaluated using the lower extremity domain of the Fugl-Meyer Assessment (FM-LE), given the established relationship between lower extremity motor impairment and balance performance following stroke (Arya et al., 2014). Participants were assigned to Rasch-derived balance ability categories, and FM-LE scores were compared across categories.

Normality of FM-LE scores within each balance ability category was assessed using the Shapiro–Wilk test prior to inferential analyses. Because the data were not normally distributed, differences among balance ability categories were examined using the Kruskal–Wallis test. When significant overall differences were identified, post hoc pairwise comparisons were performed using the Mann–Whitney U test with Bonferroni adjustment for multiple comparisons. Evidence supporting known-groups validity was defined as statistically significant differences in FM-LE scores across the Rasch-derived balance ability classifications, together with a progressive increase in FM-LE scores across successively higher balance ability levels.

Effect sizes were estimated using epsilon-squared (ε^2^). Following conventional guidelines, values of approximately 0.01, 0.06, and 0.14 were interpreted as representing small, moderate, and large effects, respectively (Tomczak and Tomczak, 2014; Field, 2013).

## 3 Results

A total of 335 individuals with stroke participated in the study (Table 1), including participants in the acute, subacute, and chronic stages of recovery, with the majority in the subacute phase. Participants ranged in age from 27 to 82 years, most had experienced an ischemic stroke, and lower extremity motor function was generally well-preserved, as reflected by the FM-LE scores.

**TABLE 1:** Demographic and clinical characteristics of participants with stroke (*n* = 335).

Characteristics	Mean (SD)	Range
Age, years	57.00 (11.58)	27–82
Sex: male/female, *n*	199/136	-
Time since stroke, days		
All stages (*n* = 335)	462 (1,130)	1–7,200
Acute (*n* = 60)	3 (2)	1–7
Subacute (*n* = 207)	33 (18)	8–118
Chronic (*n* = 68)	2172 (1,624)	126–7,200
Type of stroke: I/H, *n*	287/48	–
Affected side (right/left), *n*	180/155	–
MMSE (/30)	27.67 (2.06)	24–30
FM-LE (/34)	22.40 (10.00)	0–34
BESTest (/108)	67.96 (30.18)	0–108
S-BESTest (/39)	21.60 (12.20)	0–39

SD, standard deviation; I, ischemic; H, hemorrhage; MMSE, Mini-Mental State Examination; FM-LE, the lower extremity motor function domain of the Fugl-Meyer Assessment; BESTest, Balance Evaluation Systems Test; S-BESTest, Stroke-Balance Evaluation Systems Test.

Neither the BESTest nor the S-BESTest demonstrated significant floor or ceiling effects. Fewer than 15% of participants achieved either the minimum or maximum possible scores on both instruments. Specifically, 1.2% and 1.5% of participants obtained the minimum score on the BESTest and S-BESTest, respectively, whereas 0.9% and 3.0% achieved the maximum score.

The data were suitable for exploratory factor analysis, with high Kaiser–Meyer–Olkin values for the BESTest (0.96) and S-BESTest (0.94) and significant Bartlett’s tests of sphericity (both *p* < 0.001). Parallel analysis supported a three-factor solution for the BESTest and a two-factor solution for the S-BESTest (Tables 2, 3). The most interpretable solutions were obtained using orthogonal (Varimax) rotation.

**TABLE 2:** Three-factor structure of the 36-item Balance Evaluation Systems Test (BESTest) identified by exploratory factor analysis.

Item	Factor 1	Factor 2	Factor 3
23 Compensatory stepping correction-lateral (paretic)	**0.84**	0.06	0.34
22 Compensatory stepping correction-backward	**0.83**	0.18	0.33
17 Alternate stair touching	**0.81**	0.40	0.19
21 Compensatory stepping correction-forward	**0.81**	0.17	0.35
28 Eyes closed, foam surface	**0.79**	0.31	0.07
15 Stand on one leg (paretic)	**0.76**	0.25	0.12
34 Step over obstacle	**0.75**	0.39	0.15
14 Rise to toes	**0.75**	0.36	0.28
27 Eyes open, foam surface	**0.73**	0.47	0.13
24 Compensatory stepping correction-lateral (non-paretic)	**0.72**	0.13	0.38
32 Walk with head turns-horizontal	**0.72**	0.41	0.20
3 Ankle strength& range	**0.70**	0.24	0.25
11 Functional reach lateral (paretic)	**0.68**	0.44	0.28
35 Timed up and go	**0.68**	0.51	0.27
30 Gait-level surface	**0.68**	0.57	0.32
33 Walk with pivot turns	**0.66**	0.57	0.28
5 Sit on floor and stand up	**0.66**	0.46	0.11
4 Hip/trunk lateral strength	**0.63**	0.45	0.06
36 Timed up and go with dual task	**0.62**	0.49	0.12
16 Stand on one leg (non-paretic)	**0.59**	0.51	0.17
26 Eyes closed, firm surface	0.38	**0.77**	0.39
29 Incline eyes closed	0.41	**0.75**	0.34
25 Eyes open, firm surface	0.27	**0.74**	0.52
18 Standing arm raise	0.36	**0.71**	0.44
19 In place response-forward	0.39	**0.69**	0.50
20 In place response-backward	0.42	**0.68**	0.47
31 Change in speed	0.55	**0.67**	0.31
12 Functional reach lateral (non-paretic)	0.37	**0.61**	0.47
13 Sit to stand	0.20	**0.57**	0.49
10 Functional reach forward	0.54	**0.56**	0.45
2 Center of mass alignment	0.43	**0.55**	0.15
7 Lateral lean (non-paretic)	0.14	0.35	**0.86**
6 Lateral lean (paretic)	0.19	0.34	**0.85**
9 Verticality (non-paretic)	0.23	0.41	**0.81**
8 Verticality (paretic)	0.28	0.42	**0.81**
1 Base of support	0.14	0.21	**0.44**

Major factor loadings are shown in bold.

**TABLE 3:** Two-factor structure of the 13-item Stroke-Balance Evaluation Systems Test (S-BESTest) identified by exploratory factor analysis.

Item	Factor 1	Factor 2
15 Stand on one leg (paretic)	**0.83**	0.23
14 Rise to toes	**0.78**	0.41
23 Compensatory stepping correction-lateral (paretic)	**0.77**	0.28
27 Eyes open, foam surface	**0.76**	0.45
32 Walk with head turns-horizontal	**0.74**	0.43
4 Hip/trunk lateral strength	**0.71**	0.38
36 Timed up and go with dual task	**0.67**	0.43
16 Stand on one leg (non-paretic)	**0.67**	0.48
18 Standing arm raise	0.34	**0.87**
26 Eyes closed, firm surface	0.40	**0.85**
29 Incline eyes closed	0.41	**0.84**
12 Functional reach lateral (non-paretic)	0.32	**0.82**
31 Change in speed	0.53	**0.75**

Major factor loadings are shown in bold.

To determine whether these exploratory factors represented independent latent constructs, principal component analysis of Rasch residuals (PCAR) and disattenuated correlations were subsequently performed (Supplementary Tables 1, 2). For the S-BESTest, the Rasch measures explained 64.8% of the raw variance, and the first residual contrast had an eigenvalue of 1.95, supporting essential unidimensionality. For the BESTest, the Rasch measures explained 74.6% of the raw variance (74.3% expected), and the first residual contrast had an eigenvalue of 3.10, accounting for only 2.2% of the total variance (8.6% of the unexplained variance). Although this eigenvalue slightly exceeded the conventional criterion of 2.0, the residual contrasts declined gradually without evidence of a dominant secondary dimension. Consistent with these findings, disattenuated correlations between person measures derived from the EFA-derived item clusters were high for both the BESTest (0.92–1.05) and the S-BESTest (1.00 after bounding at unity), indicating that the apparent factors reflected a common underlying construct rather than independent dimensions.

Table 4 presents the Rasch item difficulty estimates and fit statistics for the BESTest and S-BESTest. The item hierarchy reflected progressively greater balance demands across both instruments. High-level balance tasks, including the Timed Up and Go with dual task, standing on one leg, and standing on foam with eyes closed, were among the most difficult items, whereas postural alignment and base-of-support tasks were among the easiest. A similar hierarchy was observed for the S-BESTest. Most items demonstrated acceptable fit to the Rating Scale Model. For the S-BESTest, all 13 items demonstrated infit and outfit MnSq values within 0.5–1.5. For the BESTest, four items showed infit outside this range and eleven showed outfit outside this range; two items exceeded the criterion on both statistics. All BESTest items had positive point-measure correlations (0.44–0.87). Elevated outfit values were concentrated primarily among the easiest, off-target items. All items were retained.

**TABLE 4:** Rasch item difficulty estimates and fit statistics for the BESTest and S-BESTest.

Items	BESTest				S-BESTest			
	Measure	Standard error	Infit MnSq	Outfit MnSq	Measure	Standard error	Infit MnSq	Outfit MnSq
Timed up and go with dual task	2.22	0.08	1.58	1.68	1.48	0.08	1.39	1.44
Eyes closed, foam surface	2.18	0.08	0.81	0.63	–	–	–	–
Stand on one leg (paretic)	2.18	0.08	0.71	0.74	1.44	0.08	0.67	0.78
Hip/trunk lateral strength	1.49	0.08	0.86	0.80	0.72	0.08	0.88	0.87
Compensatory stepping correction-lateral (paretic)	1.41	0.08	1.07	0.80	0.64	0.08	1.24	1.03
Stand on one leg (non-paretic)	1.37	0.08	1.19	1.19	0.60	0.08	1.06	1.17
Ankle strength and range	1.37	0.08	0.89	1.67	–	–	–	–
Walk with head turns-horizontal	1.36	0.08	1.37	1.09	0.59	0.08	1.26	1.07
Functional reach lateral (paretic)	1.23	0.08	0.83	1.08	–	–	–	–
Alternate stair touching	1.19	0.08	0.53	0.44	–	–	–	–
Step over obstacle	1.14	0.08	0.80	0.62	–	–	–	–
Compensatory stepping correction-lateral (non-paretic)	1.08	0.08	1.22	0.90	–	–	–	–
Walk with pivot turns	1.02	0.08	0.57	0.74	–	–	–	–
Sit on floor and stand up	0.99	0.08	1.41	1.14	–	–	–	–
Compensatory stepping correction-backward	0.95	0.08	1.05	0.81	–	–	–	–
Gait-level surface	0.92	0.08	0.46	0.55	–	–	–	–
Rise to toes	0.91	0.08	0.75	0.65	0.13	0.08	0.80	0.83
Timed up and go	0.89	0.08	0.60	0.52	–	–	–	–
Eyes open, foam surface	0.86	0.08	1.05	0.83	0.07	0.08	0.97	0.84
Functional reach forward	0.70	0.08	0.69	1.74	–	–	–	–
Functional reach lateral (non-paretic)	0.67	0.08	0.76	0.80	-0.12	0.08	0.82	1.03
Compensatory stepping correction-forward	0.56	0.08	1.24	0.87	–	–	–	–
Change in speed	0.15	0.09	0.88	0.69	−0.66	0.09	0.89	0.71
Standing arm raise	−0.63	0.10	1.26	1.00	−1.51	0.11	1.27	1.10
Incline eyes closed	−0.72	0.10	1.15	0.73	−1.61	0.11	1.13	0.69
Center of mass alignment	−0.77	0.10	1.41	2.31	–	–	–	–
Eyes closed, firm surface	−0.87	0.10	1.17	0.92	−1.78	0.11	1.24	0.95
In place response-backward	−1.29	0.11	1.00	0.67	–	–	–	–
In place response-forward	−1.64	0.12	0.92	0.44	–	–	–	–
Eyes open, firm surface	−1.89	0.12	1.11	0.66	–	–	–	–
Sit to stand	−2.09	0.13	1.79	1.37	–	–	–	–
Verticality (paretic)	−2.38	0.13	1.09	2.04	–	–	–	–
Verticality (non-paretic)	−2.52	0.14	1.19	1.56	–	–	–	–
Lateral lean (paretic)	−3.52	0.17	1.36	1.78	–	–	–	–
Lateral lean (non-paretic)	−3.69	0.17	1.28	1.57	–	–	–	–
Base of support	−4.84	0.23	1.78	8.55	–	–	–	–

BESTest, Balance Evaluation Systems Test; S-BESTest, Stroke- Balance Evaluation Systems Test.

Person fit was also examined (Supplementary Figures 1, 2). For the BESTest, 37 of 328 participants with non-extreme scores (11.3%) had outfit MnSq ≥ 2.0, and 9 (2.7%) had infit MnSq ≥ 2.0. For the S-BESTest, 12 of 320 participants with non-extreme scores (3.8%) had outfit MnSq ≥ 2.0, and 7 (2.2%) had infit MnSq ≥ 2.0. Mean person infit/outfit MnSq values were 1.02/1.17 for the BESTest and 1.00/0.96 for the S-BESTest. No participants were excluded on the basis of person fit.

The BESTest demonstrated a person separation index of 5.09 and person reliability of 0.96, corresponding to 7.12 statistically distinguishable strata. The S-BESTest demonstrated a person separation index of 2.88 and person reliability of 0.89, corresponding to 4.17 statistically distinguishable strata. Candidate classification boundaries were subsequently verified using the Rasch raw score-to-measure conversion table. All adjacent category midpoints were separated by more than 3 SEM, ranging from 4.0 to 5.6 SEM for the BESTest and 3.5 to 4.0 SEM for the S-BESTest (Supplementary Table 3), and person measures increased monotonically across categories. Based on this verification, six balance ability levels were established for the BESTest and four for the S-BESTest (Table 5).

**TABLE 5:** Rasch-based raw score-to-measure conversion and proposed balance ability classification levels for the Balance Evaluation Systems Test (BESTest) and Stroke- Balance Evaluation Systems Test (S-BESTest).

BESTest				S-BESTest			
Score	Measure	Standard error	Ability strata	Score	Measure	Standard error	Ability strata
0	−7.47	1.80	Very severe impairment	0	−5.22	1.83	Very severe impairment
1	−6.30	1.00		1	−4.00	1.01	
2	−5.59	0.73		2	−3.27	0.73	
3	−5.13	0.63		3	−2.83	0.61	
4	−4.78	0.56		4	−2.50	0.54	
5	−4.49	0.52		5	−2.23	0.50	
6	−4.23	0.49		6	−2.00	0.47	
7	−4.01	0.46		7	−1.79	0.45	
8	−3.81	0.44		8	−1.60	0.43	
9	−3.62	0.42		9	−1.42	0.41	
10	−3.45	0.41		10	−1.26	0.40	Severe impairment
11	−3.29	0.39		11	−1.10	0.39	
12	−3.14	0.38		12	−0.95	0.38	
13	−3.00	0.37		13	−0.81	0.38	
14	−2.87	0.36		14	−0.67	0.37	
15	−2.74	0.35		15	−0.53	0.36	
16	−2.62	0.34		16	−0.40	0.36	
17	−2.50	0.34		17	−0.28	0.36	
18	−2.39	0.33	Severe impairment	18	−0.15	0.35	
19	−2.28	0.33		19	−0.03	0.35	
20	−2.18	0.32		20	0.10	0.35	Moderate impairment
21	−2.07	0.32		21	0.22	0.35	
22	−1.98	0.31		22	0.34	0.35	
23	−1.88	0.31		23	0.46	0.35	
24	−1.79	0.30		24	0.59	0.35	
25	−1.69	0.30		25	0.71	0.36	
26	−1.61	0.30		26	0.84	0.36	
27	−1.52	0.29		27	0.97	0.37	
28	−1.43	0.29		28	1.11	0.38	
29	−1.35	0.29		29	1.26	0.39	
30	-1.27	0.28		30	1.41	0.40	Mild impairment
31	−1.19	0.28		31	1.57	0.41	
32	−1.11	0.28		32	1.75	0.43	
33	−1.04	0.27		33	1.95	0.46	
34	−0.96	0.27		34	2.17	0.49	
35	−0.89	0.27		35	2.44	0.54	
36	−0.82	0.27	Moderately severe impairment	36	2.77	0.61	
37	−0.75	0.26		37	3.21	0.73	
38	−0.68	0.26		38	3.95	1.02	
39	−0.62	0.26		39	5.18	1.84	
40	−0.55	0.25		–	–	–	–
41	−0.49	0.25		–	–	–	–
42	−0.42	0.25		–	–	–	–
43	−0.36	0.25		–	–	–	–
44	−0.30	0.24		–	–	–	–
45	−0.24	0.24		–	–	–	–
46	−0.19	0.24		–	–	–	–
47	−0.13	0.24		–	–	–	–
48	−0.07	0.24		–	–	–	–
49	−0.02	0.23		–	–	–	–
50	0.04	0.23		–	–	–	–
51	0.09	0.23		–	–	–	–
52	0.14	0.23		–	–	–	–
53	0.19	0.23		–	–	–	–
54	0.25	0.23	Moderate impairment	–	–	–	–
55	0.30	0.22		–	–	–	–
56	0.35	0.22		–	–	–	–
57	0.40	0.22		–	–	–	–
58	0.45	0.22		–	–	–	–
59	0.50	0.22		–	–	–	–
60	0.54	0.22		–	–	–	–
61	0.59	0.22		–	–	–	–
62	0.64	0.22		–	–	–	–
63	0.69	0.22		–	–	–	–
64	0.74	0.22		–	–	–	–
65	0.79	0.22		–	–	–	–
66	0.84	0.22		–	–	–	–
67	0.88	0.22		–	–	–	–
68	0.93	0.22		–	–	–	–
69	0.98	0.22		–	–	–	–
70	1.03	0.22		–	–	–	–
71	1.08	0.22		–	–	–	–
72	1.13	0.22	Moderately mild impairment	–	–	–	–
73	1.18	0.23		–	–	–	–
74	1.23	0.23		–	–	–	–
75	1.29	0.23		–	–	–	–
76	1.34	0.23		–	–	–	–
77	1.39	0.23		–	–	–	–
78	1.45	0.23		–	–	–	–
79	1.50	0.24		–	–	–	–
80	1.56	0.24		–	–	–	–
81	1.62	0.24		–	–	–	–
82	1.67	0.24		–	–	–	–
83	1.74	0.25		–	–	–	–
84	1.80	0.25		–	–	–	–
85	1.86	0.25		–	–	–	–
86	1.93	0.26		–	–	–	–
87	1.99	0.26		–	–	–	–
88	2.06	0.27		–	–	–	–
89	2.14	0.27		–	–	–	–
90	2.21	0.28	Very mild impairment	–	–	–	–
91	2.29	0.28		–	–	–	–
92	2.37	0.29		–	–	–	–
93	2.46	0.30		–	–	–	–
94	2.55	0.31		–	–	–	–
95	2.64	0.31		–	–	–	–
96	2.75	0.32		–	–	–	–
97	2.85	0.34		–	–	–	–
98	2.97	0.35		–	–	–	–
99	3.10	0.37		–	–	–	–
100	3.24	0.39		–	–	–	–
101	3.40	0.41		–	–	–	–
102	3.58	0.44		–	–	–	–
103	3.79	0.48		–	–	–	–
104	4.04	0.53		–	–	–	–
105	4.35	0.60		–	–	–	–
106	4.78	0.73		–	–	–	–
107	5.51	1.01		–	–	–	–
108	6.73	1.83		–	–	–	–

Figures 1, 2 illustrate the known-groups validity of the proposed balance ability classifications using FM-LE scores. Because FM-LE scores were not normally distributed across the classification groups (Shapiro–Wilk, all *p* < 0.05), non-parametric analyses were performed. For the BESTest, the Kruskal–Wallis test demonstrated significant differences in FM-LE scores across the six balance ability classifications [χ^2^(5) = 295.99, *p* < 0.001, ε^2^ = 0.884]. FM-LE scores increased progressively from the very severe to the very mild impairment groups. Post hoc Mann–Whitney U tests with Bonferroni correction (α = 0.0033) demonstrated significant differences between all pairwise comparisons (all adjusted *p* < 0.001).

**FIGURE 1:**
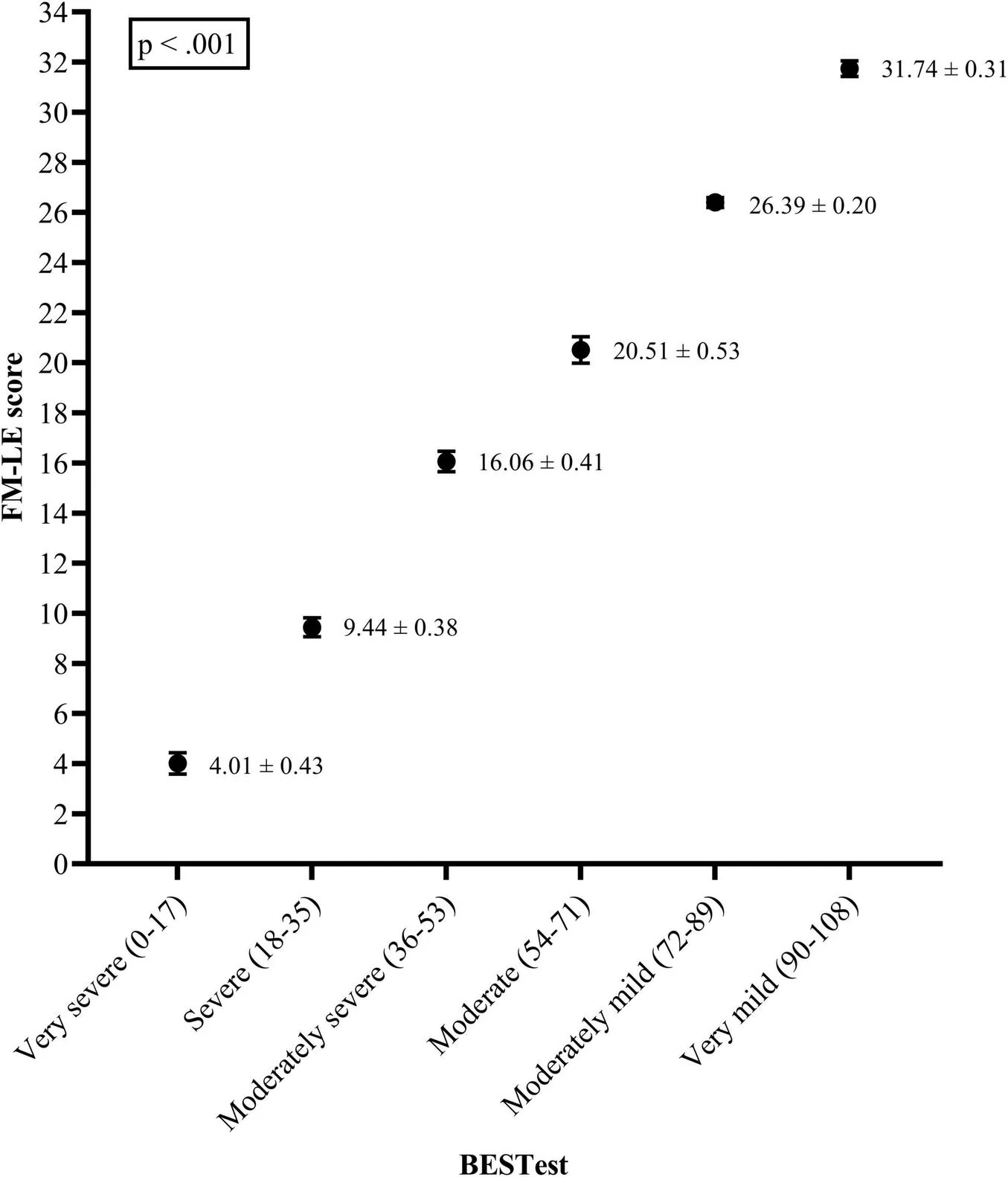
Distribution of lower extremity Fugl-Meyer Assessment (FM-LE) scores across the six Rasch-derived balance ability classifications of the BESTest. Values are presented as mean ± standard error (SE) for visualization. Statistical comparisons were performed using the Kruskal–Wallis test followed by Bonferroni-adjusted Mann–Whitney U tests.

**FIGURE 2:**
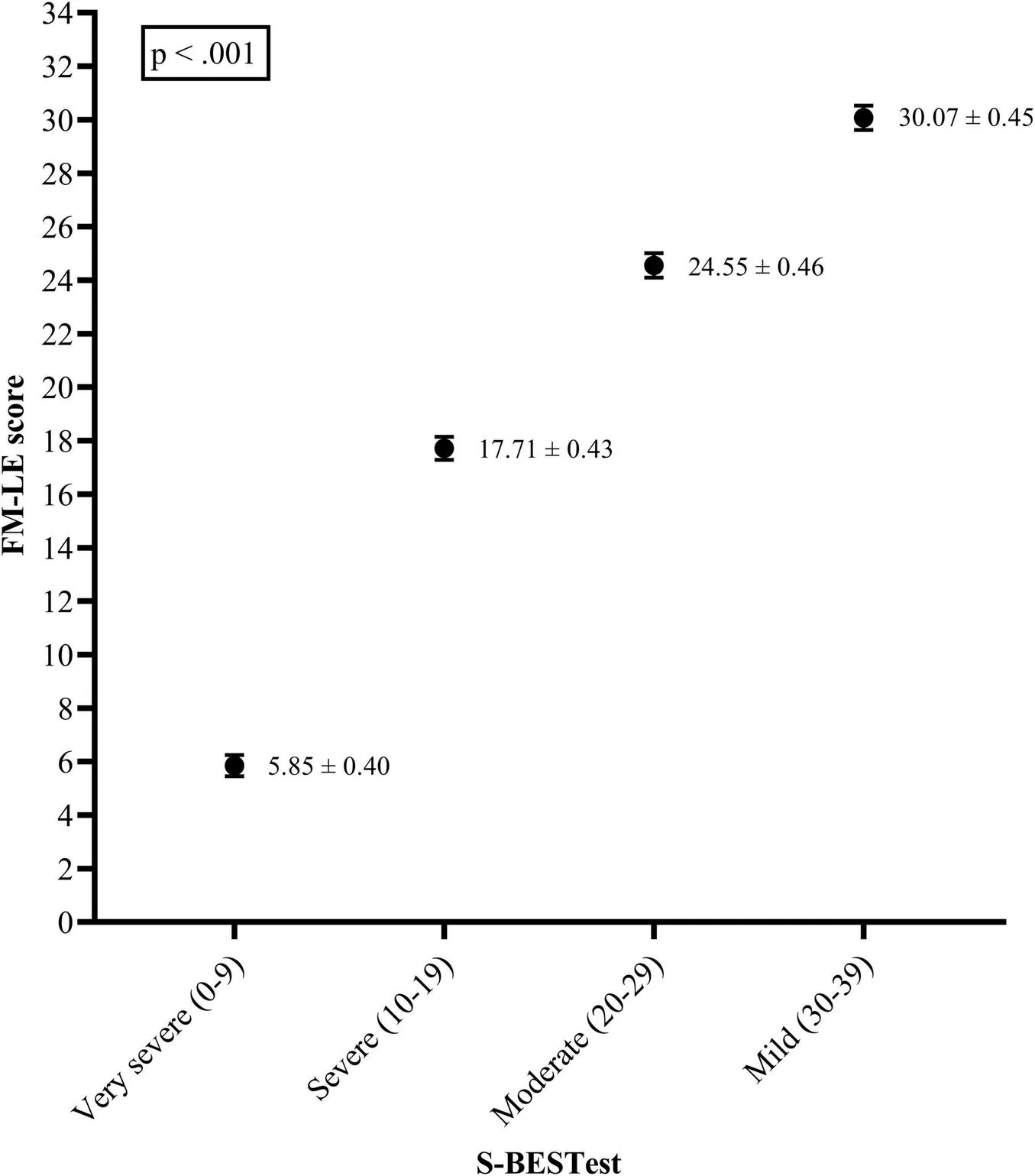
Distribution of lower extremity Fugl-Meyer Assessment (FM-LE) scores across the four Rasch-derived balance ability classifications of the S-BESTest. Values are presented as mean ± standard error (SE) for visualization. Statistical comparisons were performed using the Kruskal–Wallis test followed by Bonferroni-adjusted Mann–Whitney U tests.

Similarly, significant differences were observed across the four S-BESTest balance ability classifications [χ^2^(3) = 268.96, *p* < 0.001, ε^2^ = 0.804]. FM-LE scores increased progressively from the very severe to the mild impairment groups. *Post hoc* Mann–Whitney U tests with Bonferroni correction (α = 0.0083) also demonstrated significant differences between all pairwise comparisons (all adjusted *p* < 0.001).

## 4 Discussion

This study established Rasch-derived balance ability classifications for the BESTest and S-BESTest and demonstrated that both instruments function as essentially unidimensional measures in individuals with stroke. A major strength of the study was the large and clinically diverse sample (*n* = 335), spanning the acute, subacute, and chronic stages of stroke recovery. The proposed classifications provide a standardized framework for translating raw scores into clinically interpretable balance ability levels, which may facilitate communication among clinicians, guide individualized rehabilitation planning, and support research using meaningful rather than arbitrary score categories.

Exploratory factor analysis initially suggested a three-factor solution for the BESTest and a two-factor solution for the S-BESTest. However, inspection of the factor loading patterns indicated that these factors reflected differences in task difficulty rather than separate underlying dimensions of balance. This interpretation was subsequently confirmed by the Rasch dimensionality analyses. For the BESTest, Factor 1 and 2 each contained items from all six domains, with more demanding tasks (e.g., compensatory stepping and foam-surface standing) consistently loading on one factor and less demanding tasks (e.g., in-place postural responses and firm-surface standing) on the other. In contrast, Factor 3 comprised only the five easiest items in the instrument (items 1 and 6–9), suggesting a difficulty factor rather than a distinct clinical construct (Supplementary Tables 4–6). Difficulty factors are a recognized artifact of applying exploratory factor analysis to ordinal data using Pearson correlations. Similar findings were observed for the S-BESTest, whose two EFA-derived factors likewise reflected task difficulty rather than separate balance constructs (Supplementary Table 7).

This interpretation is further supported by comparison with the only previous factor-analytic study of the BESTest in individuals with stroke (Miyata et al., 2018). Although Miyata et al. (2018) reported a different four-factor solution after excluding 11 items, neither study reproduced Horak et al.’s (2009) six theoretical postural control systems. Instead, both studies suggest that the observed factor structures primarily reflect task characteristics, particularly task difficulty, rather than distinct balance dimensions. The additional Rasch dimensionality analyses performed in the present study provide further support for this conclusion. Although the first residual contrast of the BESTest slightly exceeded the conventional criterion of 2.0 eigenvalue units, it accounted for only 2.2% of the total variance, the residual eigenvalues declined gradually across successive contrasts, and disattenuated correlations between person measures remained uniformly high (0.92–1.05). Collectively, these findings indicate that the EFA-derived item clusters represent minor variations within a common balance construct rather than substantively distinct latent dimensions. Accordingly, both instruments were calibrated as essentially unidimensional measures, and the proposed balance ability classifications were based on the total score of each instrument.

Our findings for the S-BESTest are highly consistent with the Rasch analysis reported by Miyata et al. (2022). The overall item difficulty hierarchy was broadly similar, with more demanding dynamic balance tasks located at the upper end of the continuum and simpler static standing tasks at the lower end. Minor differences in the ordering of individual items are likely attributable to differences in participant characteristics and the distribution of balance ability between the two calibration samples. Despite these differences, both studies demonstrated excellent person reliability (0.89), identical person separation (2.88), similar variance explained by the Rasch measures (61.6% vs. 64.8%), and first residual contrasts below 2.0 eigenvalue units, supporting the essential unidimensionality of the S-BESTest. Together with previous evidence for the Mini-BESTest (Franchignoni et al., 2010), these findings suggest that retaining Domains I and II in the S-BESTest does not introduce additional measurable dimensions but instead contributes to a single underlying balance construct.

Although Rasch person measures possess the property of specific objectivity (i.e., person ability estimates are invariant to the particular subset of items administered, provided the items measure the same latent construct), the classification thresholds proposed in this study should be interpreted with appropriate caution. These thresholds were derived from the number of statistically distinguishable strata supported by the person separation index, which depends partly on the distribution of person abilities within the calibration sample. Consequently, the proposed balance ability classifications should be regarded as provisional and applicable primarily to populations with characteristics similar to those of the present sample until they are externally validated. This sample dependency may also explain why our study identified four balance ability levels, whereas Miyata et al. (2022) reported five levels in a smaller cohort (*N* = 115) consisting exclusively of individuals in the subacute stage after stroke. Future studies should externally validate these classification thresholds in independent stroke cohorts with broader clinical characteristics and balance ability distributions to establish their generalizability.

The present study extends previous Rasch analyses of the BESTest and S-BESTest by proposing clinically interpretable balance ability classifications based on Rasch person measures. Although the person separation index for the BESTest suggested approximately seven statistically distinguishable strata, six clinically meaningful balance ability categories were ultimately adopted. This distinction reflects an important principle of Rasch measurement: the maximum number of statistically distinguishable strata provides guidance regarding measurement precision but does not necessarily determine the number of clinically useful classification levels (Tennant and Conaghan, 2007; Bond et al., 2021). Rather than relying solely on ordinal total scores, the proposed classifications were derived from transitions in Rasch person measures along the latent continuum, thereby incorporating both item difficulty and person ability. Consequently, the classifications translate interval-level Rasch measures into clinically meaningful balance ability categories that may facilitate communication among clinicians, monitoring of recovery, and guide individualized rehabilitation planning. Previous studies have highlighted the value of Rasch modeling for converting ordinal clinical scores into interval-level measures and establishing clinically meaningful hierarchies (Tennant and Conaghan, 2007; Tennant and Küçükdeveci, 2023). The present study extends these applications by providing empirically derived balance ability classifications for the BESTest and S-BESTest in individuals with stroke. To our knowledge, this is the first study to establish Rasch-derived balance ability classifications for both the BESTest and S-BESTest in individuals with stroke.

Another important finding was the absence of significant floor or ceiling effects for either instrument. Fewer than 15% of participants achieved the minimum or maximum possible scores, indicating that both the BESTest and S-BESTest were able to discriminate balance ability across a broad range of performance, from severe impairment to near-normal function (McHorney and Tarlov, 1995). These findings are consistent with previous studies reporting minimal floor and ceiling effects in individuals with stroke (Chinsongkram et al., 2014) and further support the suitability of both instruments for assessing balance across different stages of stroke recovery.

Known-groups validity analysis provided additional evidence supporting the clinical validity of the proposed Rasch-derived balance ability classifications. For both the BESTest and the S-BESTest, FM-LE scores increased progressively across higher balance ability levels, with significant differences observed between all classification categories. Together with the excellent Rating Scale Model fit, these findings indicate that the proposed classifications successfully discriminate individuals with different levels of lower extremity motor impairment. This relationship is clinically plausible because successful balance performance depends on lower extremity motor control, postural coordination, and sensorimotor integration, all of which are commonly impaired following stroke (Pollock et al., 2000; Garland et al., 2009). The findings are consistent with previous reports demonstrating strong associations between lower extremity motor recovery and balance performance after stroke (Cho et al., 2025) and support the use of the proposed classifications as clinically meaningful indicators of balance ability.

An important clinical implication of the present findings is that, although exploratory factor analysis identified multiple item groupings, subsequent Rasch analyses demonstrated that both the BESTest and the S-BESTest function as essentially unidimensional measures of balance ability. Consequently, clinicians can continue to interpret the total score while benefiting from the newly proposed Rasch-derived balance ability classifications. Because these classifications are based on interval-level person measures rather than ordinal raw scores, they provide a more standardized framework for communicating balance status, monitoring recovery, and evaluating rehabilitation outcomes. The classifications are expected to be most applicable to individuals with stroke who have characteristics similar to those of the present sample.

The Rasch-derived item hierarchy may also assist clinicians in selecting balance tasks appropriate to an individual’s current ability level. For example, individuals with severe balance impairment may initially benefit from practicing less demanding tasks before progressing to more challenging activities, whereas those with mild impairment may begin at a higher level of task difficulty. Such an approach could facilitate individualized progression of balance training, although its clinical effectiveness requires prospective evaluation. Future studies should determine whether classification-guided rehabilitation improves patient outcomes, whether transitions between balance ability levels reflect clinically meaningful recovery, and whether the proposed classification thresholds generalize to independent stroke cohorts with different clinical characteristics and ability distributions.

The fit results further support the measurement performance of the S-BESTest. All 13 S-BESTest items demonstrated acceptable infit and outfit statistics, and person misfit was observed in only 3.8% of participants. These findings indicate that the shorter instrument retained coherent measurement performance in the present sample, although direct comparison of person-fit rates with the BESTest should be interpreted cautiously because the instruments differ in length and item targeting. Together with the Rasch-derived balance ability classifications established for both instruments, these findings provide clinicians with flexibility in selecting the assessment most appropriate for their clinical setting. The BESTest may be preferable when a comprehensive evaluation of balance deficits is required, whereas the S-BESTest offers a practical and time-efficient alternative for routine clinical assessment and outcome monitoring. Because both instruments provide standardized and clinically interpretable balance ability classifications, clinicians can select the assessment according to time constraints, patient tolerance, and clinical objectives while maintaining a common framework for interpreting balance impairment. These findings complement previous studies demonstrating that shortened versions of the BESTest retain robust measurement properties while substantially reducing administration time (Franchignoni et al., 2015; Winairuk et al., 2019; Meseguer-Henarejos et al., 2025).

Several limitations should be acknowledged. First, although this study established Rasch-derived balance ability classifications for individuals with stroke, these classifications have not yet been externally validated or prospectively evaluated in routine clinical practice. Consequently, their applicability should currently be considered limited to populations with characteristics similar to those of the present sample. Future studies should validate the proposed classification thresholds in independent stroke cohorts with different clinical characteristics and ability distributions and determine whether classification-guided rehabilitation improves clinical decision-making and patient outcomes. Second, administration of the full BESTest required approximately 1 h, which may have resulted in participant fatigue and consequently influenced performance on later BESTest items, including those contributing to the extracted S-BESTest scores. Although this concern is less relevant to the S-BESTest because of its substantially shorter administration time, future studies should evaluate the influence of fatigue on assessment performance. Third, S-BESTest scores were extracted from the administration of the full BESTest rather than being obtained through independent administration. Although this approach ensured identical testing conditions for comparing the two instruments, performance during routine administration of the standalone S-BESTest may differ slightly. Therefore, independent validation of the S-BESTest classifications is warranted. Fourth, information regarding the use of assistive devices during assessment was not systematically recorded. Although the influence of gait aid use could not be evaluated directly, its effects were likely reflected, at least in part, in the gait and stepping items because the BESTest scoring criteria explicitly account for the use of assistive devices. Finally, this study did not evaluate the responsiveness of the proposed balance ability classifications to rehabilitation or their ability to predict longitudinal recovery. Future longitudinal studies should determine whether transitions between classification levels correspond to clinically meaningful improvements in balance and functional outcomes.

## 5 Conclusion

Both the BESTest and the S-BESTest functioned as essentially unidimensional measures of balance ability with acceptable Rasch measurement properties in individuals with stroke. The Rasch-derived balance ability classifications demonstrated good known-groups validity and provide a standardized framework for translating total scores into clinically meaningful balance ability levels. These classifications have the potential to improve the interpretation of balance performance, support individualized rehabilitation planning, facilitate patient stratification, and enhance longitudinal monitoring of recovery following stroke.

## Data Availability

The raw data supporting the conclusions of this article will be made available by the authors, without undue reservation.
