# Supplementary material for: Rasch-based classification of post-stroke balance ability: dimensionality and clinical validation of the BESTest and S-BESTest
Source: Front Aging Neurosci. 2026 Oct 2;18:1889177. doi: 10.3389/fnagi.2026.1889177 (PMC13672641; doi:10.3389/fnagi.2026.1889177)
Supplement: Supplementary file 1 [file Data_Sheet_1.docx]

**Table S1** **Principal component analysis of Rasch residuals (PCAR)** for each EFA-derived factor and for the full BESTest and S-BESTest.

| **Scale** | **Raw Variances explained by measured (%)** | **Unexplained variance in 1st contrast (Eigenvalue)** | **Unexplained variance in 1st contrast (%)** |
| --- | --- | --- | --- |
| Full BESTest | 74.6 | 3.10 | 2.2 |
| BESTest Factor1 | 59.8 | 3.11 | 6.2 |
| BESTest Factor 2 | 78.8 | 2.95 | 5.7 |
| BESTest Factor 3 | 88.4 | 1.83 | 4.3 |
| Full S-BESTest | 64.8 | 1.95 | 5.3 |
| S-BESTest Factor 1 | 58.9 | 1.71 | 8.8 |
| S-BESTest Factor 2 | 63.9 | 1.89 | 13.7 |

**Table S2** Observe and Disattenuated correlations between factor person measures (r) between each EFA-derived factor

| **Dimension Pair** | **Observe r** | **Disattenuate r** |
| --- | --- | --- |
| BESTest Factor 1 x Factor 2 | 0.90 | 1.05 |
| BESTest Factor 1 x Factor 3 | 0.71 | 0.92 |
| BESTest Factor 2 x Factor 3 | 0.76 | 0.92 |
| S-BESTest Factor 1 x Factor 2 | 0.88 | 1.00  (bounded at unity) |

**Table S3** Separation of Adjacent Balance Ability Classifications for the BESTest and S-BESTest

| Adjacent Classifications | Adjacent midpoint  Measures (logits) | Difference (logits) | Mean SEM | Separation SEM |
| --- | --- | --- | --- | --- |
| **BESTest** |  |  |  |  |
| Very severe → severe | −3.62 → −1.61 | 2.01 | 0.36 | 5.6 |
| Severe → moderately severe | −1.61 → −0.30 | 1.31 | 0.27 | 4.9 |
| Moderately severe → moderate | −0.30 → +0.64 | 0.94 | 0.23 | 4.1 |
| Moderate → moderately mild | +0.64 → +1.56 | 0.92 | 0.23 | 4.0 |
| Moderately mild → very mild | +1.56 → +3.10 | 1.54 | 0.31 | 5.0 |
| **S-BESTest** |  |  |  |  |
| Very severe → severe | −2.50 → −0.67 | 1.83 | 0.46 | 4.0 |
| Severe → moderate | −0.67 → +0.59 | 1.26 | 0.36 | 3.5 |
| Moderate → mild | +0.59 → +2.17 | 1.58 | 0.42 | 3.8 |

**Note.** Separation was calculated as the difference between adjacent midpoint Rasch measures divided by the mean standard error of measurement (SEM) of the two adjacent measures. Values >3 SEM indicate statistically distinguishable adjacent classification levels.

**Table S4** Mapping 3 exploratory factors of the BESTest and the 6 BESTest domains

| **Domain** | **Factor 1** | **Factor 2** | **Factor 3** |
| --- | --- | --- | --- |
| I Biomechanical constraints | 3, 4, 5 | 2 | 1 |
| II Stability limits/verticality | 11 | 10, 12 | 6, 7, 8, 9 |
| III Anticipatory postural adjustments | 14, 15, 16, 17 | 13, 18 | — |
| IV Reactive postural responses | 21, 22, 23, 24 | 19, 20 | — |
| V Sensory orientation | 27, 28 | 25, 26, 29 | — |
| VI Stability in gait | 30, 32, 33, 34, 35, 36 | 31 | — |

**Table S5 Detailed items in Factor 1 and Factor 2**

| **Domain** | **Factor 1 (harder task)** | **Factor 2 (easier task)** |
| --- | --- | --- |
| I | Ankle strength/range (3), Hip/trunk lateral strength (4), Sit on floor & stand up (5) | CoM alignment (2) |
| II | Functional reach lateral, paretic side (11) | Functional reach forward (10), lateral non-paretic (12) |
| III | Rise to toes (14), stand on paretic leg (15), non-paretic leg (16), alternate stair touching (17) | Sit to stand (13), standing arm raise (18) |
| IV | Compensatory stepping — forward, backward, lateral paretic, lateral non-paretic (21–24) | In-place response forward (19), backward (20) |
| V | Eyes open foam (27), eyes closed foam (28) | Eyes open firm (25), eyes closed firm (26), incline EC (29) |
| VI | Gait, head turns, pivot turns, obstacle, TUG, TUG dual-task (30, 32–36) | Change in gait speed (31) |

**Table S6** Difficulty rank of items in Factor 3 (The easiest)

| Difficulty rank (of 36) | Item | Measure (logits) |
| --- | --- | --- |
| 1 the easiest | 1 Base of support | −4.84 |
| 2 | 7 Lateral lean, non-paretic | −3.69 |
| 3 | 6 Lateral lean, paretic | −3.52 |
| 4 | 9 Verticality, non-paretic | −2.52 |
| 5 | 8 Verticality, paretic | −2.38 |
| 6 | 13 Sit to stand | −2.09 |

**Table S7** Mapping 2 exploratory factors of the S-BESTest and the 6 BESTest domains

| **Domain** | **Factor 1** | **Factor 2** |
| --- | --- | --- |
| I Biomechanical constraints | 4 | - |
| II Stability limits/verticality | - | 12 |
| III Anticipatory postural adjustments | 14, 15, 16 | 18 |
| IV Reactive postural responses | 23 | - |
| V Sensory orientation | 27 | 26, 29 |
| VI Stability in gait | 32, 36 | 31 |

**Figure S1** Wright map from the Rasch analysis of the BESTest

**Figure S2** Wright map from the Rasch analysis of the S-BESTest
